# A meta-analysis of intravenous thrombolysis versus bridging therapy for ischemic stroke

**DOI:** 10.1097/MD.0000000000030879

**Published:** 2022-09-30

**Authors:** Raoqiong Wang, Shuangyang Li, Linyao Hao, Zhichuan Wang, Zhengxin Ge, Sijin Yang

**Affiliations:** a National Traditional Chinese Medicine Clinical Research Base of the Affiliated Traditional Chinese Medicine Hospital of Southwest Medical University, Luzhou, China.

**Keywords:** acute ischemic stroke, intravenous thrombolysis, mechanical thrombectomy, meta-analysis

## Abstract

The purpose of this study was to perform a pooled analysis of randomized controlled trials (RCT) of intravenous thrombolysis (IVT) versus bridging therapy of intravenous thrombolysis and mechanical thrombectomy (IVMT), comparing the efficacy and safety of the two in patients with acute ischemic stroke (AIS).

**Methods:**

All eligible RCT articles from database establishment to December 8, 2021 were searched in databases such as PubMed, Ovid, Embase, Web of science, Cochrane Library, etc. Efficacy outcomes were assessed by modified RANKIN scal (mRS) score, complete recanalization or reperfusion (TICI), National Institute of Health Stroke Scal (NIHSS) score, 90-day mortality, 24 to 36 h incidence of symptomatic intracranial hemorrhage (sICH).

**Results:**

Our study included 6 RCT involving 1717 patients. The proportion of the primary efficacy outcome (mRS score 0‐2 at 90 days) was significantly different between IVT and IVMT (OR 0.51; 95% CI 0.35‐0.76). For the secondary efficacy outcome, the study found a significant difference in the proportion of TICI (pooled OR was 0.055, 95% CI 0.07‐0.33). There was a significant difference in the 24 h NIHSS score between the IVT group and the IVMT group (pooled MD was 3.25, 95% CI 0.80‐5.70). There were no significant differences in the NIHSS score at 90 days, the death rate at 90 days, and the sICH at 24 to 36 hours between the two groups.

**Conclusions:**

This study confirms that IVMT is more effective and safe than IVT alone in patients with AIS. However, more and higher-quality randomized clinical trials comparing IVMT to IV alone are warranted for validation.

## 1. Introduction

Acute ischemic stroke (AIS) is a localized ischemic necrosis or softening of brain tissue caused by ischemia and hypoxia. It has the characteristics of high morbidity, high disability and high mortality.^[1]^ Early treatment can significantly improve the prognosis of ischemic stroke and reduce disability and mortality.^[2]^ The 2021 European Stroke Organization (ESO) guidelines for intravenous thrombolysis (IVT) in AIS propose that IVT is the only systemic reperfusion therapy approved for patients with AIS, and it is also the first choice for clinical treatment of AIS.^[3]^ IVT has the characteristics of simple operation and low cost, but the time window of IVT is narrow and must be used within 4.5 hours of the onset of symptoms. It has a low rate and more bleeding, so the failure rate of IVT alone is high.^[3]^ Guidelines recommend that endovascular mechanical thrombectomy can be combined to improve the recanalization rate.^[3]^ However, so far, researchers have mainly compared the efficacy and safety of mechanical thrombectomy (MT) alone and MT combined with IVT in the treatment of acute stroke, but there is no relevant meta-analysis to confirm that IVT combined with MT is superior to IVT alone. Therefore, this meta-analysis aimed to compare the efficacy and safety of IVT alone versus bridging therapy intravenous thrombolysis and mechanical thrombectomy (IVMT) in the treatment of AIS.

## 2. Research Methods

### 2.1. Inclusion and exclusion criteria of literature

The methodology of this meta-analysis followed PRISMA guidelines.

#### 2.1.1. Inclusion criteria for the literature.

The research object is AIS, and the diagnosis of intracranial hemorrhage is excluded. The research design is RCT. The language is Chinese and English. There are clear records of IVMT. And the full text of the literature is available. The results of the study describe the modified RANKIN scale (mRS, 0‐2) score, complete recanalization or reperfusion (TICI), NIHSS score, 90-day mortality, and symptomatic intracranial hemorrhage (sICH) in the two groups of patients after treatment.

#### 2.1.2. Literature exclusion criteria.

Important baseline characteristics of the study subjects (sample size, age, gender) or important study results (mRS 0‐2) score, TICI, NIHSS score, 90-day death sICH data are lacking. Arterial thrombolysis occurred during the study. Repeated publications and unable to obtain original literature data.

### 2.2. Search strategy and research selection

PubMed, Ovid, Embase, Web of science, Cochrane Library, CBM, Wanfang data, Sino Med, VIP databases were searched for all eligible RCT articles from database creation to December 8, 2021. English uses “intravenous thrombolysis or IVT, mechanical thrombolysis or mechanical thrombectomy or MT, AIS or acute cerebral infarction” as search term; Chinese uses” intravenous thrombolysis, mechanical thrombolysis, mechanical thrombolysis, acute ischemic brain Stroke, acute ischemic stroke, acute ischemic stroke, acute cerebral infarction” were search terms, and all searches were performed using MeSH and free words.

### 2.3. Data extraction and quality assessment

Two researchers independently screened the literature in strict accordance with the inclusion and ranking criteria of the literature. After first screening the literature by reading the title and abstract, further screening by reading the full text, if there is any disagreement, it will be judged by a third party, and finally the inclusion or exclusion will be decided through discussion. Two researchers independently extracted and included relevant research data, title, author, year, country, diagnostic method of acute ischemic stroke, sample size of each group, age, gender, treatment method, treatment time, outcome indicators, evaluation methods and main findings. The authenticity of the RCTs was assessed by 2 investigators according to the Cochrane Handbook, and the risk of bias of the literature was assessed according to the Cochrane Risk of Bias Tool. In case of disagreement, a third party was consulted. The risk of bias was assessed using seven criteria, including random sequence generation, concealed assignment, blinding of participants and personnel, incomplete outcome data, selective reporting, and other bias. The risk of bias was classified into three categories: “low” (+), “high” (–), and “unclear” (?).

### 2.4. Evaluation of research results

#### 2.4.1. Main efficacy results.

The patients were followed up for functional recovery after 90 days of treatment, using the modified RANKIN scale (mRS). mRS scoring standard^[4]^: level 0: completely asymptomatic; level 1: although there are symptoms, there is no obvious dysfunction, and all daily work and life can be completed; level 2: ,mild disability, unable to complete all activities before the illness, but does not need help, can take care of their daily affairs; level 3: moderate disability, requires partial assistance, but can walk independently; level 4: moderate to severe disability, unable to walk independently, needs help from others in daily life; level 5: severe disability, bedridden, fecal incontinence, and total dependence on others in daily life; level 6: death. Therefore, when the mRS score is 0 to 2, the outcome is assessed as a better prognosis for the patient. When the mRS score was greater than 2, the outcome was assessed as poor prognosis.

#### 2.4.2. Secondary efficacy results.

First: assessment of vascular recanalization using TICI vascular perfusion grading. TICI grading standard^[5]^: level 0 (no perfusion): no antegrade blood flow distal to the vascular occlusion; level I (diffusion without perfusion): the contrast medium partially passes through the occlusion site, but cannot fill the distal vessels; level II (partial perfusion): the contrast agent completely fills the distal end of the artery, but the filling and removal speed is slower than that of the normal artery; level IIa (contrast filling < 2/3 of the blood supply area of the involved vessel); level IIb (complete filling of contrast medium, but delayed emptying); level III (complete perfusion): the contrast agent fills the distal vessels completely and rapidly, and is rapidly cleared. Therefore, TICI grades IIb to III represent better recanalization of the diseased blood vessels. Second: NIHSS (National Institute of Health Stroke Scale) score,^[6]^ which is a quantitative indicator of the severity of AIS disease, is often used as a surrogate endpoint in clinical research, and stratifies patients according to the NIHSS score to guide clinical decision-making. Effective treatment was defined as the NIHSS score decreased by more than 4 points or the symptoms disappeared completely after 24 hours of treatment. Third: statistical analysis was performed on the incidence of adverse events (mortality, sICH) in the two groups of patients. Mortality: the patient died of acute ischemic stroke after 90 days of treatment in both groups (other causes excluded). Mortality = number of deaths/total number of studies in each group. Symptomatic intracerebral hemorrhage rate (sICH) within 24 to 36 hours: intracranial hemorrhage was found by MRI and CT within 24 to 36 hours after treatment, and clinical symptoms were present. NIHSS score increased by atleast 4 points. Symptomatic intracerebral hemorrhage rate = number of sICH cases/total number of study participants in each group.

### 2.5. Data synthesis and statistical analysis

RevMan 5.3 software was used for meta-analysis. Pooled effect size: count data and measurement data were selected as odds ratio (OR), relative risk (RR), weighted mean difference (MD) or standard mean difference (standard mean difference, SMD) for statistical analysis. Heterogeneity analysis: *I*² was used to evaluate the heterogeneity of the study. For example, when *I*² < 50%, *P* > .05, it indicated that the heterogeneity was small, and a fixed effect model was used; when *I*² ≥ 50%, *P* ≤ .05, it indicates large heterogeneity. Firstly, the source of heterogeneity was analyzed. If there was statistical heterogeneity, a random effect model was used. If the heterogeneity could not be eliminated, the source of heterogeneity was analyzed from both methodological and clinical aspects, and subgroup analysis was used. A funnel plot was used to check for publication bias, and if the funnel plot was asymmetrical, it was suggested that bias may have occurred.

## 3. Research results

### 3.1. Literature screening process and search results

**Figure 1.**
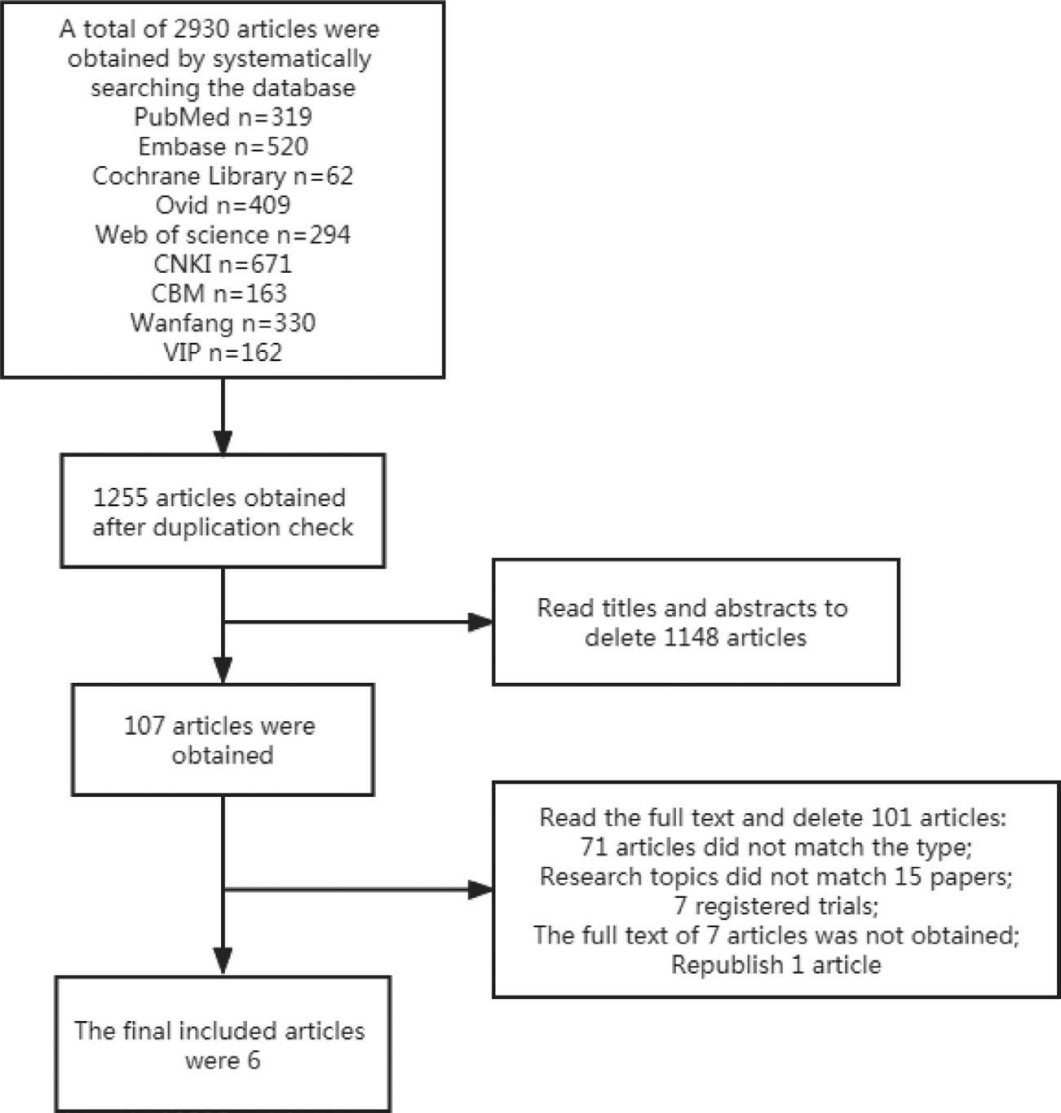
The flow chart of literature screening.

Through systematic retrieval, a total of 2930 literatures were obtained, and 1255 literatures were obtained after duplicate checking. After reading the title and abstract, 107 papers were obtained after preliminary screening, and after further reading the full text, 101 papers were deleted (71 papers did not match the type of literature; 15 papers did not match the research topic; 7 papers were registered trials; 7 papers were not obtained full text; 1 paper was published repeatedly), 6 articles with a total of 1717 patients were finally included. The specific literature screening flow chart is shown in Figure 1.

### 3.2. Basic characteristics of the included studies

Six RCTs were included, published between 2013 and 2016, with a total of 1717 patients (745 patients with IVT; 969 patients with intravenous thrombolysis combined with mechanical thrombectomy; 3 patients withdrew after randomization). The basic characteristics of each study are shown in Table 1. The baseline characteristics of the included studies are shown in Table 2.

**Table 1:** Basic characteristics of each study.

Research	Nation	Time	Diagnosis method	Sample size	IVT:IVT + MT	Healing period	Outcomes
Bracard 2016	France	2010.6-2015.2	CT/MRA	414	1:1	Intravenous thrombolysis < 4h Mechanical thrombectomy < 5h	①The proportion of mRS ≤ 2 points at 3 months after intervention; ②NIHSS scores at 24h, 7d and 3 months after intervention; ③Barthel score and quality of life at 3 months after intervention; ④90-day mortality;⑤24-hour cerebral hemorrhage.
Broderick 2013	USA, Canada, Australia, Europe	2006.8-2012.4	CT	656	1:2	Intravenous thrombolysis < 3 hours; Mechanical thrombectomy < 5 hours	①Proportion of mRS ≤ 2 points at 90 days after intervention; ② 90-day mortality rate; ③ 30-hour cerebral hemorrhage; ④ 90-day stroke recurrence rate
Campbell 2015	Australia, New Zealand	2012.8-2014.10	CT	70	1:1	Intravenous thrombolysis < 4.5 hours; Mechanical thrombectomy < 6h	①Reperfusion ratio; ②NIHSS score at 1 to 3 days; ③mRS score at 90 days; ④90-day mortality rate; ⑤ Symptoms of intracranial hemorrhage
Goyal 2015	Canada, United States, South Korea, Ireland, United Kingdom	2013.2-2014.10	CT	316	1:1	Intravenous thrombolysis < 4.5 hours; Mechanical thrombectomy not specified	① 90-day mRS score; ② early recanalization and reperfusion; ③ intracranial hemorrhage; ④ angiographic complications; ⑤ 90-day neurological dysfunction and death.
Muir 2016	U.K	2013.4-2015.4	CT/MRA	65	1:1	Intravenous thrombolysis < 4.5 hours; Mechanical thrombectomy < 6 hours	① 90-day mRS score; ② NIHSS score improvement at 24 hours after intervention; ③ time at home (time from stroke onset to day 90 in usual residence); ④ mortality rate;
Saver 2015	America, Europe	2012.12-2014.11	CT/MRI	196	1:1	Intravenous thrombolysis not specified; Mechanical thrombectomy < 6 hours	① 90-day mRS score; ② 90-day mortality; ③ 27-hour intracranial hemorrhage symptoms

IVT = intravenous thrombolysis, ICH = symptomatic intracranial hemorrhage, IVMT = intravenous thrombolysis and mechanical thrombectomy, MT = mechanical thrombectom, mRS = modified RANKIN scale, NIHSS =National Institute of Health Stroke Scales, TICI = complete recanalization or reperfusion.

**Table 2:** Baseline characteristics of included studies.

Sociodemographic data	IVT, n/N (%) or mean ± SD	IVMT, n/N (%) or mean ± SD
Sample size	745	969
Sex (male)	388/745 (52.1)	489/969 (50.5)
Age	67.5 ± 12.8/745	67.9 ± 12.7/969
Baseline characteristics		
NIHSS	16.3 ± 2.6/710	17.1 ± 3.9/934
ASPECTS (8‐10)	268/462	367/671
Comorbidities		
Hypertension	493/744	624/965
Diabetes	157/744	169/969
Hyperlipidemia	221/411	295/613
Coronary heart disease	113/517	142/727
Smoking	87/358	89/359
Blockage		
ICA	65/337	52/332
M1	254/337	258/332
M2	14/337	17/332

ICA = internal carotid artery, IVT = intravenous thrombolysis, IVMT = intravenous thrombolysis and mechanical thrombectomy, MT = mechanical thrombectom, M1 = M1 segment of middle cerebral artery (MCA), M2 = M2 segment of middle cerebral artery (MCA).

### 3.3. Quality assessment and risk of bias of included studies

**Figure 2.**
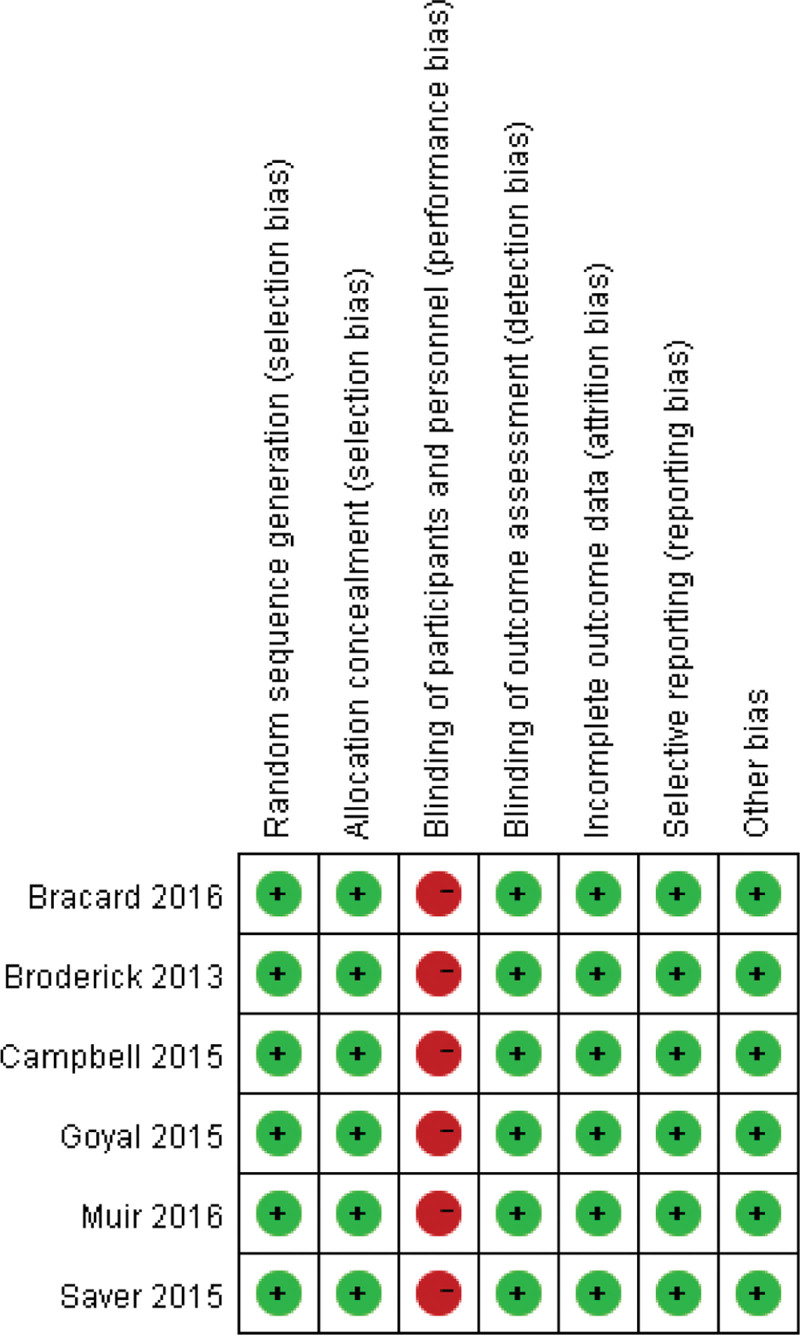
Literature risk of bias map.

Due to the particularity of the intervention measures, the six included RCTs were rated as “high risk of bias” in the evaluation of “blinding research subjects and interventionists”, and the rest of the items were rated as “low risk of bias”. The detailed results are shown in Table 3, and the risk of bias map is shown in Figure 2.

**Table 3:** Evaluation of literature quality of included studies.

Research	Generation of random sequences	Allocation hiding for random sschemes	Blinding study subjects and interventionists	Blinding of outcome assessors	Completeness of outcome measures	Possibility of selective reporting of findings	Other bias
Bracard2016	Low risk of bias	Low risk of bias	High risk of bias	Low risk of bias	Low risk of bias	Low risk of bias	Low risk of bias
Broderick2013	Low risk of bias	Low risk of bias	High risk of bias	Low risk of bias	Low risk of bias	Low risk of bias	Low risk of bias
Campbell2015	Low risk of bias	Low risk of bias	High risk of bias	Low risk of bias	Low risk of bias	Low risk of bias	Low risk of bias
Goyal2015	Low risk of bias	Low risk of bias	High risk of bias	Low risk of bias	Low risk of bias	Low risk of bias	Low risk of bias
Muir2016	Low risk of bias	Low risk of bias	High risk of bias	Low risk of bias	Low risk of bias	Low risk of bias	Low risk of bias
Saver2015	Low risk of bias	Low risk of bias	High risk of bias	Low risk of bias	Low risk of bias	Low risk of bias	Low risk of bias

## 4. Meta-analysis results

### 4.1. Comparison of mRS (0–2 points) scores after 90 days of treatment for AIS between the two groups

**Figure 3.**
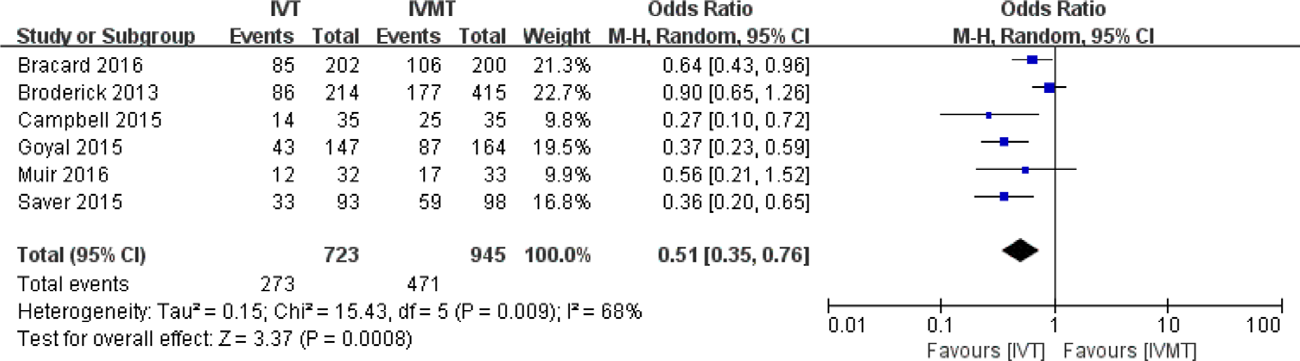
Meta-analysis of the proportion of mRS scores 0 to 2 at 90 days.

The proportion of mRS score 0 to 2 at 90 days in the IVT group was slightly lower than that in the IVMT group. The combined effect size of the random effect model showed that there was a significant difference between the IVT group and the IVMT group in the proportion of mRS scores 0 to 2 at 90 days (pooled OR 0.51, 95% CI 0.35, 0.76, *P* .001). The results are shown in Figure 3.

### 4.2. Comparison of TICI between the two groups in the treatment of AIS

**Figure 4.**
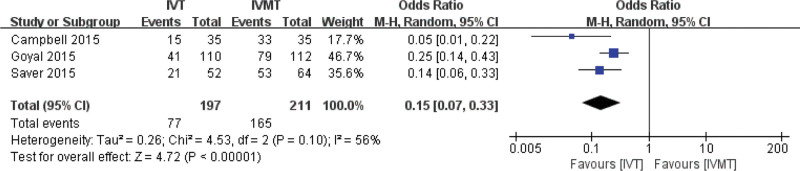
Meta-analysis of the proportion of recanalization or reperfusion.

The proportion of TICI in the IVMT group was slightly higher than that in the IVT group. The combined effect size of the random effect model showed that there was a significant difference in the proportion of TICI between the IVT group and the IVMT group (pooled OR 0.055, 95% CI 0.07, 0.33, *P* < .001). The results are shown in Figure 4.

### 4.3. Comparison of 24 hours NIHSS scores in the treatment of AIS between the two groups

**Figure 5.**
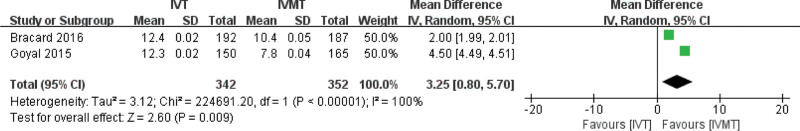
Meta-analysis of 24 hours NIHSS scores (National Institute of Health Stroke Scales).

The combined effect size of the random effect model showed that there was a significant difference in the NIHSS scores between the IVT group and the IVMT group at 24 hours (pooled MD 3.25, 95% CI 0.80, 5.70, *P* = .009). The results are shown in Figure 5

### 4.4. Comparison of 24 hours NIHSS scores in the treatment of AIS between the two groups

**Figure 6.**
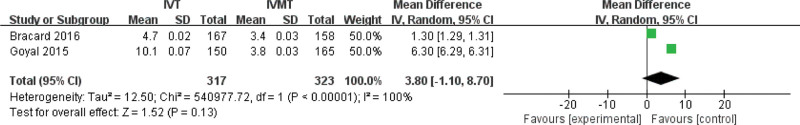
Meta-analysis of 90 days NIHSS scores (National Institute of Health Stroke Scales).

The combined effect size of the random effect model showed that there was no significant difference in the NIHSS scores between the IVT group and the IVMT group at 90 days (pooled MD 3.80, 95% CI -1.10, 8.70, *P* = .053). The results are shown in Figure 6

### 4.5. Comparison of 90-day mortality of AIS between the two groups

**Figure 7.**
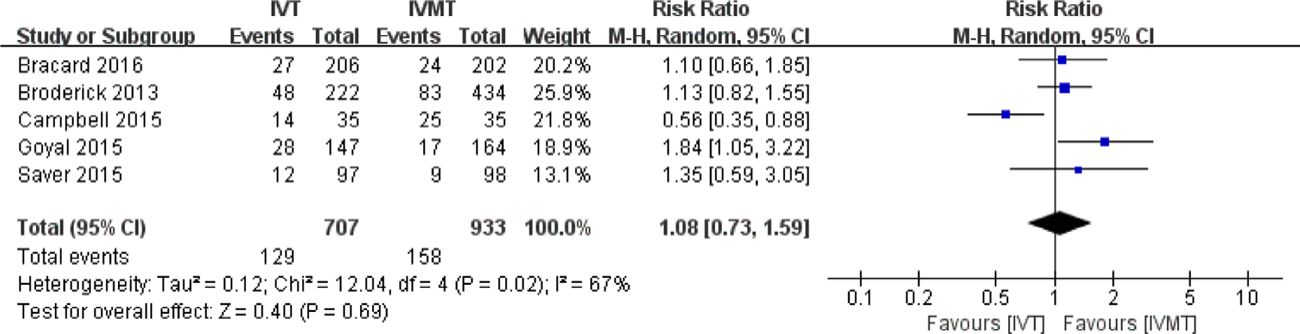
Meta-analysis of 90-days mortality.

The combined effect size of the random effect model showed that there was no significant difference in the proportion of deaths at 90 days between the IVT group and the IVMT group (pooled RR 1.08, 95% CI 0.73, 1.59, *P* = .69). The results are shown in Figure 7.

### 4.6. Comparison of the two groups in the treatment of AIS with sICH within 24 to 36 hours

**Figure 8.**
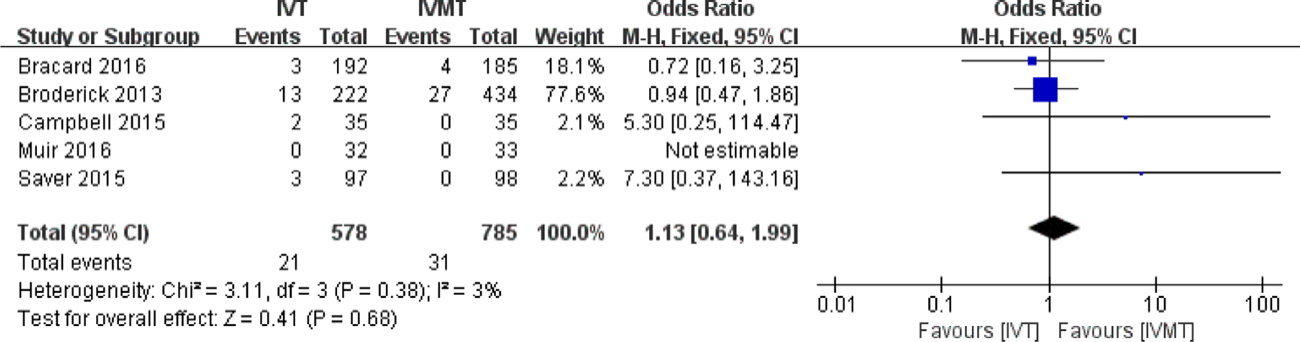
Meta-analysis of symptomatic intracranial hemorrhage at 24 to 36 hours.

The combined effect size of the fixed effect model showed that there was no significant difference in the incidence of sICH between the IVT group and the IVMT group at 24 to 36 hours (pooled OR 1.13, 95% CI 0.64, 1.99, *P* = .68). The results are shown in Figure 8.

## 5. Discussion

Acute ischemic stroke refers to brain tissue necrosis caused by sudden interruption of cerebral blood supply.^[7]^ It is usually mainly due to atherosclerosis and thrombosis of the arteries supplying blood to the brain, which narrows or even occludes the lumen, resulting in focal acute cerebral insufficiency. Early restoration of blood perfusion in the ischemic brain region is the key to clinical treatment of acute cerebral infarction.^[8]^ In terms of treatment, the latest guidelines recommend IVT to improve functional outcomes in patients with acute ischemic stroke within 4.5 hours of symptom onset. This treatment method has the advantages of simple operation and low cost, but the recanalization rate is relatively low.^[3]^ With the improvement of minimally invasive interventional treatment technology, the application of endovascular mechanical thrombectomy technology has improved the recanalization rate of cerebral infarction and shortened the recanalization time. However, intravascular mechanical thrombectomy is easy to loosen the thrombus, causing tiny thrombus to block the small blood vessels at the distal end, affecting the therapeutic effect.^[9]^ Therefore, whether the combined application of IVMT can benefit better has attracted widespread clinical attention.

The mRS score is a scale used to evaluate the state of neurological recovery in stroke patients. As an important indicator for predicting the prognosis of stroke, mRS score is a recognized clinical efficacy endpoint in cerebrovascular disease research.^[10]^ A key aspect of successful recanalization (TICI) is the restoration of cerebral blood flow. In clinical trials, this is often measured by the TICI scale, which assesses recanalization by digital subtraction angiography.^[11]^ The results of this study showed that compared with IVT alone and intravenous thrombolysis combined with mechanical thrombectomy, IVMT improved the mRS score and the success rate of vascular recanalization. It is proved that IVMT can better improve the neurological outcome of patients with acute ischemic stroke and obtain better clinical therapeutic effect than IVT. There was no significant difference in the NIHSS scores 90 days after treatment between the two groups. However, the NIHSS score at 24 hours after treatment in the IVMT group was significantly lower than that in the IVT group, indicating that IVMT can improve the neurological function of patients. There was no significant difference in 90-day mortality and 24 to 36 hours sICH adverse events between the two groups, indicating that intravenous thrombolysis combined with mechanical thrombectomy did not increase the safety risk.

In conclusion, intravenous thrombolysis combined with mechanical thrombectomy in the treatment of ACI patients can improve the vascular recanalization rate, reduce the NIHSS and mRS scores, and does not increase the safety risk, and the effect is better than that of IVT alone. However, this meta-analysis has some limitations: there are few literatures included in this study; the data is not ideal and the heterogeneity is too high; the literature included in this study did not clearly analyze whether the efficacy and safety of the two groups were different due to different specific parts of thrombus, and there was confounding bias. Further large sample RCT should be carried out, which is worthy of further discussion.

This study was supported by Sichuan Academy of traditional Chinese medicine reserve candidate project (Number: Sichuan Chinese Medicine Letter (2020) No. 85).The authors have no conflicts of interest to disclose.

## Author contributions

**Data curation:** Linyao Hao, Raoqiong Wang, Shuangyang Li.

**Formal analysis:** Zhengxin Ge, Zhichuan Wang.

**Validation:** Zhengxin Ge, Zhichuan Wang.

**Writing – original draft:** Raoqiong Wang, Shuangyang Li.

**Writing – review & editing:** Sijin Yang.

## References

[1] Ho JP. Acute ischemic stroke: emergency department management after the 3-hour window. Emerg Med Pract. 2021;23(Suppl 6):1–33. 34133111

[2] He G Wei L Lu H Li Y Zhao Y Zhu Y. Advances in imaging acute ischemic stroke: evaluation before thrombectomy. Rev Neurosci. 2021;32:495–512. 33600678 10.1515/revneuro-2020-0061

[3] Berge E Whiteley W Audebert H. European Stroke Organisation (ESO) guidelines on intravenous thrombolysis for acute ischaemic stroke. Eur Stroke J. 2021;6:I–LXII. 10.1177/2396987321989865 PMC7995316 33817340

[4] Saver JL Chaisinanunkul N Campbell BCV. XIth stroke treatment academic industry roundtable. Standardized nomenclature for modified rankin scale global disability outcomes: consensus recommendations from stroke therapy academic industry roundtable XI. Stroke. 2021;52:3054–62. 34320814 10.1161/STROKEAHA.121.034480

[5] Liebeskind DS Bracard S Guillemin F. eTICI reperfusion: defining success in endovascular stroke therapy. J Neurointerv Surg. 2019;11:433–8. 30194109 10.1136/neurintsurg-2018-014127

[6] Yamal JM Grotta JC. National Institutes of Health stroke scale as an outcome measure for acute stroke trials. Stroke. 2021;52:142–3. 33317412 10.1161/STROKEAHA.120.032994

[7] Herpich F Rincon F. Management of acute ischemic stroke. Crit Care Med. 2020;48:1654–63. 32947473 10.1097/CCM.0000000000004597 PMC7540624

[8] Silva GS Nogueira RG. Endovascular treatment of acute ischemic stroke. Continuum (Minneap Minn). 2020;26:310–31. 32224754 10.1212/CON.0000000000000852

[9] Krishnan R Mays W Elijovich L. Complications of mechanical thrombectomy in acute ischemic stroke. Neurology. 2021;97(20 Suppl 2):S115–25. 34785610 10.1212/WNL.0000000000012803

[10] Lee SY Kim DY Sohn MK. Determining the cut-off score for the modified barthel index and the modified rankin scale for assessment of functional independence and residual disability after stroke. PLoS One. 2020;15:e0226324. 31995563 10.1371/journal.pone.0226324 PMC6988933

[11] Seo WK Nam HS Chung JW. TAB-TICI Score: successful recanalization score after endovascular thrombectomy in acute stroke. Front Neurol. 2021;12:692490. 34721254 10.3389/fneur.2021.692490 PMC8551570

